# Narcolepsy: Pathophysiology, Diagnosis, Management, and Future Directions, a Narrative Review

**DOI:** 10.1002/brb3.71116

**Published:** 2025-12-07

**Authors:** Natasha Elaine Hastings, Khalil El Abdi, Fazeela Bibi, Bilal Aslam, Milka Rosario Nuñez, Muhammad Tahla Rehman Sherani, Sujeet Shadmani, Umama Alam, Vohra Maham Hassan, Hoor‐e‐Ainaa, Hania Imran, James Hanna, Said Hamid Sadat

**Affiliations:** ^1^ School of Medicine St. George's University St. George's Grenada; ^2^ Faculty of Medicine and Pharmacy of Rabat Mohammed V University Rabat Morocco; ^3^ Jinnah Medical and Dental College Karachi Pakistan; ^4^ University of Lahore Lahore Pakistan; ^5^ Universidad Iberoamericana (UNIBE) Santo Domingo Dominican Republic; ^6^ Army Medical College Rawalpindi Pakistan; ^7^ Shaheed Mohtarma Benazir Bhutto Medical College Karachi Pakistan; ^8^ Khyber Medical College Khyber Pakistan; ^9^ Ayub Medical College Abbotabad Pakistan; ^10^ Wah Medical College Wah Cantt Pakistan; ^11^ Kabul University of Medical Sciences Abu Ali Ibn Sina Kabul Afghanistan

**Keywords:** cataplexy, excessive daytime sleepiness, hypocretin, narcolepsy, therapy

## Abstract

**Objective:**

To critically synthesize the current understanding of narcolepsy's pathophysiology, diagnostic challenges, and treatment landscape, and to articulate the emerging paradigm shift from symptomatic management toward disease modification.

**Data Sources:**

This narrative review is based on a strategic search of literature from PubMed and Google Scholar published between 2015 and 2025, focusing on disease mechanisms, diagnosis, and established and emerging therapeutic interventions.

**Synthesis of Findings:**

Narcolepsy is a neurological disorder fundamentally driven by an irreversible loss of hypocretin‐producing neurons, a process strongly linked to an autoimmune response in individuals with the HLA‐DQB1*0602 allele. Despite this well‐defined pathophysiology, diagnosis is often delayed by nearly a decade due to limited physician awareness and symptom overlap with a wide range of psychiatric and metabolic comorbidities. Current pharmacological strategies, while providing partial relief from excessive daytime sleepiness and cataplexy, are purely symptomatic and fail to address the core hypocretin deficiency. The field is now at a critical inflection point, with promising research into hypocretin receptor agonists, targeted immunotherapies, and neuromodulation techniques poised to directly address the underlying pathology.

**Conclusion and Relevance:**

The existing therapeutic approach to narcolepsy is palliative, not restorative, leaving a significant unmet need for interventions that can alter the disease's natural history. The future of narcolepsy management depends on translating novel pathophysiological insights into disease‐modifying therapies. Achieving this requires a concerted effort to accelerate diagnosis, validate new clinical endpoints, and prioritize the development of interventions capable of restoring neurological function. Such an approach holds the potential to move beyond symptom suppression and fundamentally improve the lives of individuals with narcolepsy.

## Graphical abstract

This visual summary outlines the pathophysiology of narcolepsy, driven by hypocretin loss, and details its clinical diagnosis, current management strategies, and the shift toward future disease‐modifying therapies like orexin agonists and immunomodulation.

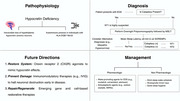

**Abbreviations**
AASM: American Academy of Sleep Medicine
ADHD: Attention Deficit Hyperactive Disorder
CBT: Cognitive Behavioral Therapy
CBT‐N: Cognitive‐Behavioral Treatment for Narcolepsy
CRH: Corticotropin‐Releasing Hormone
CSF: Cerebrospinal Fluid
DBS: Deep Brain Stimulation
EAN: European Academy of Neurology
EDS: Excessive Daytime Sleepiness
EMA: European Medicines Agency
FDA: U.S. Food and Drug Administration
GHB: Gamma‐Hydroxybutyrate
HCRU: High Healthcare Resource Utilization
HLA: Human Leukocyte Antigen
IVIG: Intravenous Immunoglobulin
MBSR: Mindfulness‐Based Stress Reduction
MSLT: Multiple Sleep Latency Test
NT1: Narcolepsy Type 1
NT2: Narcolepsy Type 2
OSA: Obstructive Sleep Apnea
PLMs: Periodic Limb Movements
PSG: Polysomnography
RBD: REM Sleep Behavior Disorder
SNRIs: Serotonin‐Norepinephrine Reuptake Inhibitors
SOREMPs: Sleep‐Onset REM Periods
SSRIs: Selective Serotonin Reuptake Inhibitors
TMS: Transcranial Magnetic Stimulation

## 1 Introduction

Narcolepsy is a life‐long, debilitating sleep disorder. The hallmarks of this disease are excessive daytime sleepiness (EDS), cataplexy, disrupted sleep, hallucinations, and sleep paralysis (Scammell 2015). There are two primary kinds of narcolepsy: Type 1 (NT1) and Type 2 (NT2). NT1 involves episodes of cataplexy, which is when patients suffer from sudden muscle weakness, and/or a deficiency in hypocretin‐1, a neuropeptide originating from the hypothalamus, in their cerebrospinal fluid (CSF) (Weaver et al. 2018). Comparatively, patient's with NT2 have normal levels of hypocretin in their CSF and they do not suffer from cataplexy symptoms (Weaver et al. 2018). The unifying symptom of both types is EDS (Scammell 2015).

Limited awareness of narcolepsy and its symptoms among physicians, along with the misattribution of symptoms to other diseases or comorbidities, often leads to diagnostic delays of eight years or more (3). Symptoms often present between ages of 10 and 25, with peak presentation occurring at 15 years old (Zhang et al. 2022). This holds true more often than not, but is not to say that cataplexy cannot, and does not, present at any age. The loss of hypocretin, produced from the hypothalamus, has been identified as a main contributor to the development of narcolepsy (Mignot et al. 1997). It has been presumed that an immune‐mediated destruction of neurons producing hypocretin occurs, and is linked to an HLA subtype identified as DQB1*0602 (Vaarala et al. 2014).

Global prevalence of narcolepsy varies due to different diagnostic criteria (Rosenthal et al. 2021) and population demographics, with estimates ranging from 25 to 50 per 100,000 people (Wang et al. 2022). A large study across North America, Europe, and South Korea estimated a combined prevalence of 42.4 per 100,000, with Narcolepsy Type 1 (NT1) at 19.1 and Type 2 (NT2) at 23.3 per 100,000 (Ohayon et al. 2025). Disparities are notable across ethnicities; in Japan, estimates range from a prevalence of 37.5 per 100,000 to as high as 0.59% of the population, with an incidence of 5.1 per 100,000 person‐years (Kadotani et al. 2025). Narcolepsy significantly reduces health‐related quality of life (HRQoL) (Tadrous et al. 2021) and is linked to high healthcare resource utilization (HCRU) (Giertz et al. 2025). In the United States, individuals with narcolepsy have approximately twice the number of inpatient admissions, emergency visits, and prescriptions as controls (Doane et al. 2025). This elevated economic and clinical burden underscores the need for timely diagnosis and effective management.

This narrative review aims to examine current interventions for narcolepsy management, including pharmacological and non‐pharmacological approaches, while identifying gaps in existing research. In addition, it explores emerging therapies and future directions to enhance treatment strategies and improve the quality of life for individuals with narcolepsy.

## 2 Methodology of Research

This narrative review was conducted through a systematic search of the PubMed and Google Scholar databases. The primary literature search focused on publications from 2015 to 2025 to capture the most current and relevant evidence. In addition, seminal publications from 2000 to 2015 were included to ensure comprehensive coverage of foundational research, especially on hypocretin biology, HLA associations, and core diagnostic criteria.

Search terms included “narcolepsy,” “hypocretin,” “therapeutics,” “compliance,” “artificial intelligence,” “sleep hygiene,” “cognitive behavioral therapy,” and “epidemiology.” The review encompassed psychiatric comorbidities such as anxiety, depression, attention deficit hyperactivity disorder (ADHD), and schizophrenia, as well as medical and neurological conditions including obesity, cardiovascular disease, stroke, and sleep apnea, categorizing these appropriately to avoid terminology errors.

**FIGURE 1:**
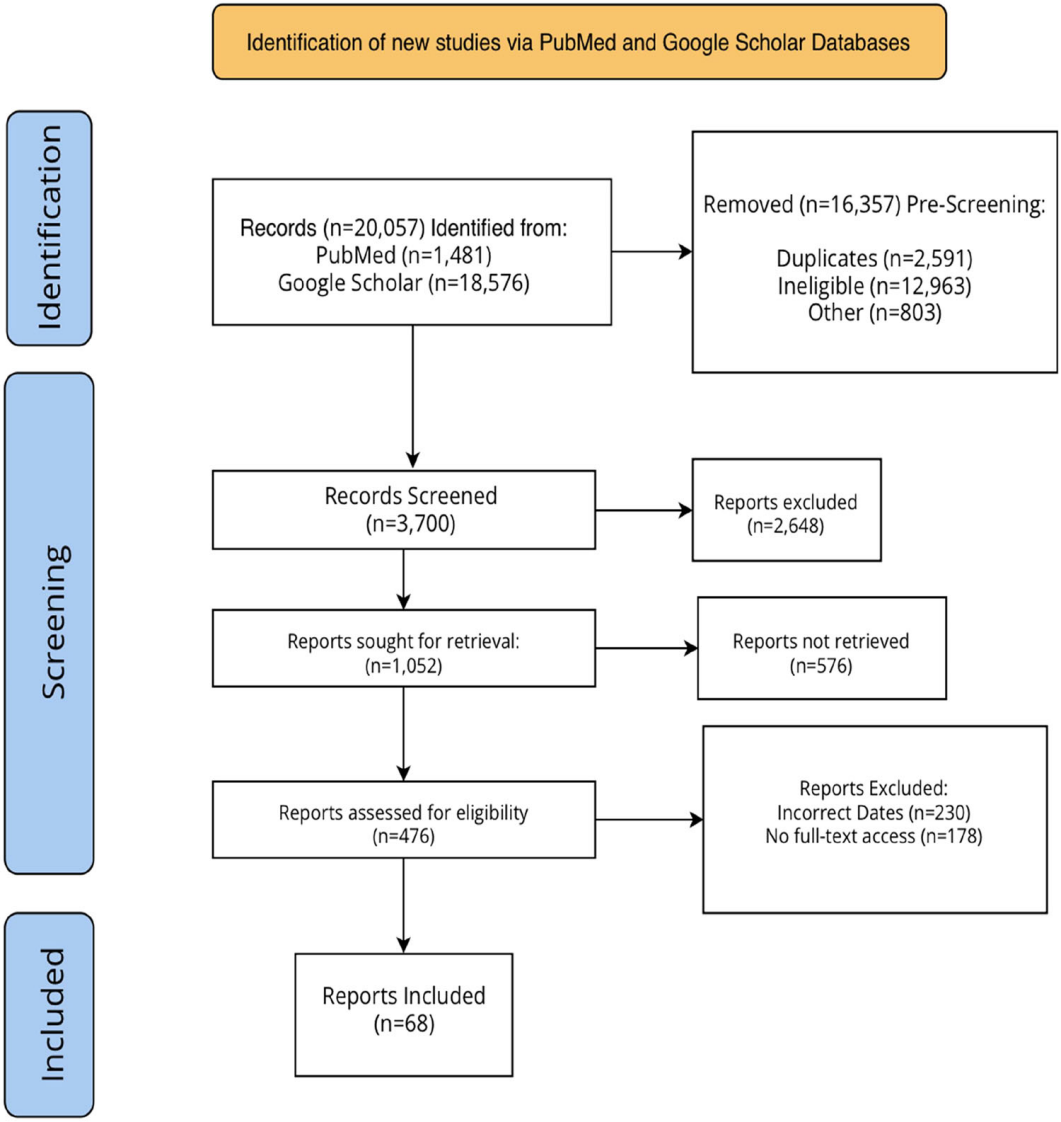
PRISMA flowchart showing article selection process.

Titles and abstracts of identified articles were screened for relevance, followed by full‐text review of articles meeting inclusion criteria: English language, original articles or reviews published between 2000 and 2025. No limitations were placed on study design or population to maintain narrative comprehensiveness. The study selection process is summarized in the PRISMA flow diagram (Figure 1).

## 3 Pathophysiology and Clinical Presentation of Narcolepsy

Narcolepsy is characterized by five primary symptoms: EDS, cataplexy, sleep paralysis, interrupted nighttime sleep, and hallucinations (Pelayo and Lopes 2006). Among these, EDS is nearly universal, presenting as sudden, uncontrollable urges to sleep, often described as “sleep attacks,” that do not improve with adequate nighttime sleep (Zeman et al. 2004). Cataplexy, a pathognomonic feature of NT1, affects approximately 60%–70% of patients (Bassetti et al. 2019). It is triggered by strong emotions and results in episodes of sudden muscle weakness, ranging from mild muscle slackening to complete collapse while maintaining consciousness (Mirabile and Sharma 2025). Sleep paralysis occurs in 25%–50% of patients with narcolepsy and is marked by a temporary inability to move or speak while falling asleep or awakening (Cheung et al. 2017). Hypnagogic (at sleep onset) or hypnopompic (during awakening) hallucinations are reported in 33%–80% of individuals (Cheung et al. 2017). Fragmented nighttime sleep, characterized by frequent awakenings, is seen in 30%–95% of patients (Roth et al. 2013). Beyond these core symptoms, additional manifestations include REM sleep behavior disorder (RBD), periodic limb movements (PLMs), automatic behavior without conscious awareness, and compulsive eating disorders (Thorpy and Dauvilliers 2015; Jennum et al. 2013; Zorick et al. 1979; Palaia et al. 2011; Chabas et al. 2007).

The pathophysiology of narcolepsy is most strongly linked to a deficiency in hypocretin, a neuropeptide that regulates wakefulness (Chow and Cao 2016). In NT1, hypocretin deficiency is typically caused by the loss of hypocretin‐producing neurons in the hypothalamus, potentially triggered by an autoimmune reaction (Malenka et al. 2009). Genetic predispositions also play a significant role, with HLA‐DQB1*0602 being a strongly associated allele (Shan et al. 2022; John et al. 2013). Other genetic factors, including CPT1B, CHKB, and TRIB2 autoantibodies, have also been implicated (Kish et al. 1992; Luo et al. 2018; Miyagawa et al. 2008). In addition, environmental triggers such as infections may contribute to autoimmune‐mediated neuronal loss (Cvetkovic‐Lopes et al. 2010; Faraco et al. 2013).

In contrast, the pathophysiology of narcolepsy type 2 (NT2) is less well understood and considered a more heterogeneous condition (Cavalli et al. 2025). By definition, individuals with NT2 do not experience cataplexy and have normal or unmeasured hypocretin levels in their CSF (Bassetti et al. 2019). Diagnosis of NT2 is largely one of exclusion, as no specific biomarkers have been identified (Son et al. 2021). Current research suggests that NT2 may represent a spectrum of disorders, possibly including cases with partial hypocretin deficiency that do not meet the threshold for NT1 or dysfunction in downstream hypocretin signaling pathways (Huth et al. 2024).

Finally, secondary narcolepsy can arise from brain injuries, tumors, or other neurological conditions that damage the sleep‐wake regulatory regions of the brain (Yi et al. 2015). Emerging evidence also suggests that other neurotransmitter systems—including histamine, dopamine, and serotonin—may modulate the severity and variability of narcolepsy symptoms (Wing et al. 2011; Hor et al. 2011).

## 4 Narcolepsy and Comorbidity

Narcolepsy is a complex neurological disorder characterized not only by its primary sleep‐related symptoms but also by a wide range of medical and psychiatric comorbidities that complicate diagnosis and management (Gudka et al. 2022).

Psychiatric conditions are among the most common, with studies showing that anxiety disorders affect 21%–53% of patients, and depression occurs in 27%–57% (Chen et al. 2025). These mood disorders are often present at diagnosis and are associated with poorer clinical outcomes, including an increased risk for suicidal ideation (Chen et al. 2025). In pediatric populations, symptoms of ADHD are notably prevalent, particularly in NT2, impairing academic and social function (Lecendreux et al. 2015). In addition, a small subset (3%–4%) of patients with NT1 experience psychotic‐like symptoms, such as vivid hallucinations (Wang et al. 2016), which are thought to result from REM sleep intrusions rather than a primary psychotic disorder (Hanin et al. 2021).

Metabolic and cardiovascular dysfunction are also highly prevalent (Mohammadi et al. 2021). Rapid weight gain often follows the onset of NT1, which is attributed to hypothalamic dysregulation that reduces the basal metabolic rate (Ponziani et al. 2016; Bosco et al. 2018). This contributes to high rates of obesity, with children diagnosed with narcolepsy having a significantly higher body mass index than their peers (Jervis et al. 2025). Consequently, patients with narcolepsy face elevated cardiovascular risks, including higher rates of hypertension, dyslipidemia, and major adverse cardiovascular events (Kaufmann et al. 2025). Many NT1 patients exhibit a non‐dipping nocturnal blood pressure pattern, a known risk factor for stroke (Dauvilliers et al. 2012).

Furthermore, other comorbid conditions significantly impact patients' quality of life. Obstructive sleep apnea (OSA) is frequently diagnosed in individuals with narcolepsy—with a prevalence ranging from 25% to over 50%—creating a complex diagnostic and management challenge (Miano et al. 2024). Chronic pain disorders, including migraines and fibromyalgia, are also commonly reported (Dauvilliers et al. 2011). Finally, significant cognitive impairment is a core feature of the condition, with meta‐analyses confirming that patients with both NT1 and NT2 exhibit moderate‐to‐large deficits in attention and executive function (Harel et al. 2024). These far‐reaching comorbidities underscore the need for an integrated, multidisciplinary approach to care.

## 5 Diagnosis and Assessment of Narcolepsy

**FIGURE 2:**
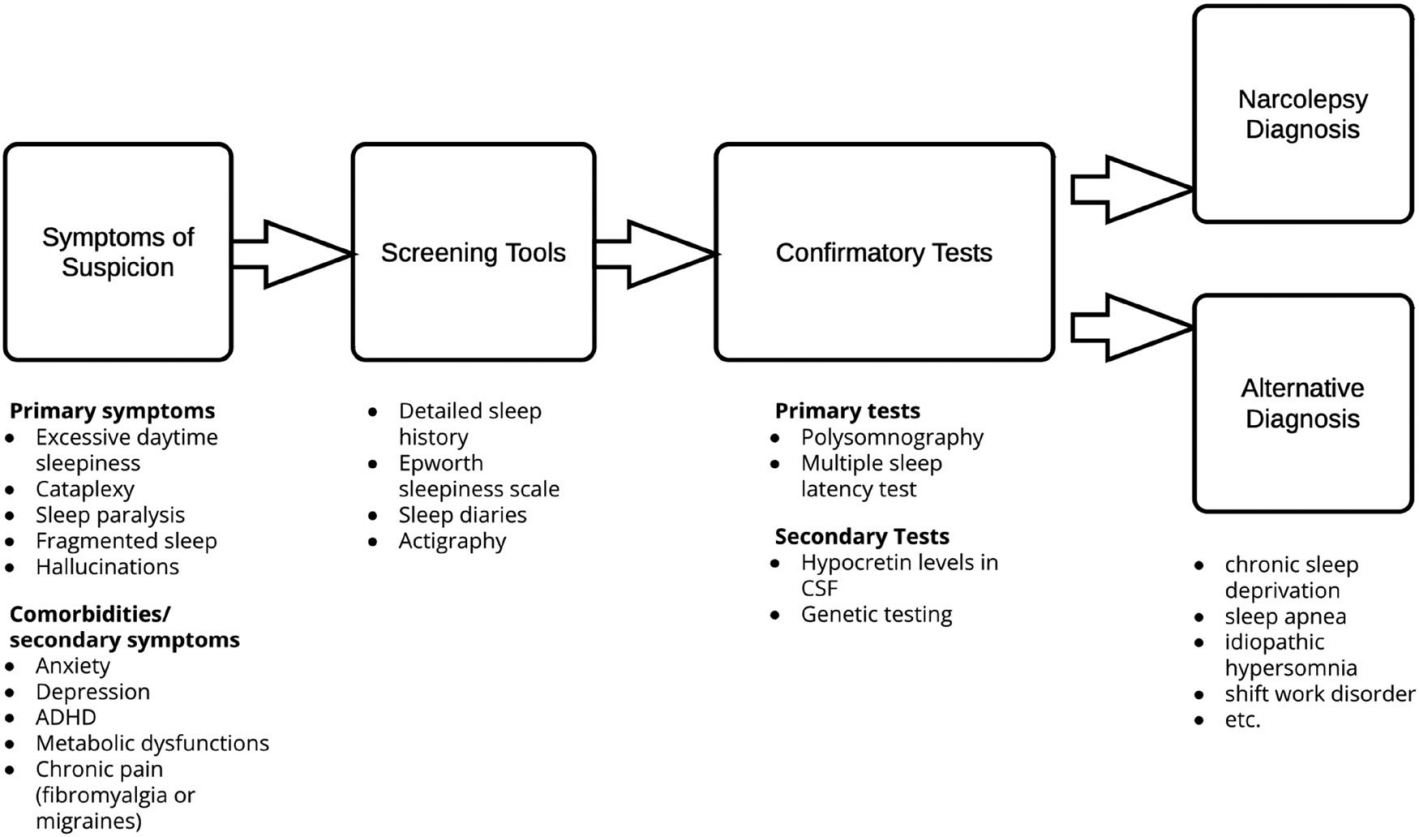
Clinical pathway of diagnosis of narcolepsy.

The diagnosis of narcolepsy is a multifaceted process (summarized in Figure 2) that integrates a detailed clinical history with objective testing, guided by criteria from the International Classification of Sleep Disorders, Third Edition (ICSD‐3) and the Diagnostic and Statistical Manual of Mental Disorders, Fifth Edition (DSM‐5) (Ruoff and Rye 2016). The evaluation begins with a comprehensive sleep history and the use of validated screening tools like the Epworth Sleepiness Scale and the Swiss Narcolepsy Scale, the latter of which shows high sensitivity and specificity for identifying NT1 (Zub et al. 2024).

The cornerstone of objective diagnosis involves an in‐laboratory, overnight video‐polysomnography (PSG) followed by a Multiple Sleep Latency Test (MSLT) the next day (Torstensen et al. 2023). The preceding PSG is mandatory to rule out other causes of EDS, such as OSA or PLM disorder, and to ensure adequate sleep duration, as sleep deprivation can invalidate MSLT results (Zhang et al. 2021). A key finding on the PSG is a sleep‐onset REM period (SOREMP), defined as REM sleep occurring within 15 min of sleep onset, which is highly suggestive of narcolepsy (Reiter et al. 2015).

The MSLT, which consists of five scheduled nap opportunities, measures the patient's physiological sleep tendency and propensity for REM sleep dysregulation (Arand and Bonnet 2019). A mean sleep latency of ≤8 min combined with ≥2 SOREMPs is the classic diagnostic threshold for narcolepsy (Goddard et al. 2021). However, the MSLT has significant limitations, including poor test‐retest reliability, particularly for NT2 and idiopathic hypersomnia (Trotti 2020). Its results can be confounded by factors such as chronic sleep deprivation, circadian rhythm disorders, and the use of REM‐suppressing medications like antidepressants (Krahn et al. 2021).

For NT1, two highly specific biomarkers can confirm the diagnosis. The definitive marker is a low level of hypocretin‐1 in the CSF, with a value ≤110 pg/mL being diagnostic. While highly accurate, this test is invasive and typically reserved for atypical cases (Mignot et al. 2002). In addition, genetic testing for the HLA‐DQB1*06:02 allele is strongly associated with NT1 (present in over 90% of cases) and can support the diagnosis, though it is not diagnostic on its own due to its presence in a significant portion of the general population (Capittini et al. 2018).

The diagnostic criteria from the ICSD‐3‐TR and DSM‐5‐TR differ slightly in their approach (Ruoff and Rye 2016). The ICSD‐3‐TR formally distinguishes between NT1 (requiring either cataplexy or low CSF hypocretin) and NT2 (EDS and MSLT findings without cataplexy and with normal or unmeasured hypocretin) (Ruoff and Rye 2016). The DSM‐5‐TR, often considered more practical for clinical use, requires EDS plus at least one of the following: Cataplexy, hypocretin deficiency, or characteristic PSG/MSLT findings (including an nSOREMP) (Ruoff and Rye 2016). A crucial part of the diagnostic process is the differential diagnosis, which includes idiopathic hypersomnia, chronic sleep deprivation, and other sleep or psychiatric disorders that can cause EDS (Nyhuis and Fernandez‐Mendoza 2023).

## 6 Current Interventions

The cornerstone of narcolepsy management consists of non‐pharmacological and behavioral interventions, which are universally recommended (Bassetti et al. 2021). This foundational layer begins with robust patient education regarding the chronic nature of the disorder, followed by the implementation of a strict sleep‐wake schedule to stabilize circadian rhythms (Chakravorty and Rye 2003). Strategically timed diurnal naps, typically 15–20 min in duration, are highly encouraged as they can effectively and temporarily restore alertness (Bassetti et al. 2021; Mullington and Broughton 1993). These core strategies are supplemented by established principles of sleep hygiene, including the avoidance of evening stimulants like caffeine (Bhattarai and Sumerall 2017). Furthermore, specialized psychological interventions such as Cognitive Behavioral Therapy for narcolepsy (CBT‐N) and mindfulness‐based practices are emerging as valuable adjuncts to help patients manage the profound psychosocial burden of the disease and improve sleep quality (Franceschini et al. 2021).

Pharmacologic intervention, which is layered upon this behavioral framework, is meticulously tailored to address the primary symptom clusters of EDS and REM sleep dysregulation, including cataplexy (Franceschini et al. 2021). For the management of EDS, several first‐line agents with distinct mechanisms of action are recommended. Modafinil and its R‐enantiomer, armodafinil, promote wakefulness primarily through dopamine reuptake inhibition (Loland et al. 2012). Solriamfetol, a more recent dopamine and norepinephrine reuptake inhibitor, has demonstrated robust efficacy in clinical trials (Subedi et al. 2020). A mechanistically novel, non‐stimulant option is pitolisant, which functions as a histamine H3 receptor antagonist/inverse agonist to enhance histaminergic neurotransmission, a key wake‐promoting pathway (Hirano et al. 2022). Sodium oxybate, including a lower‐sodium formulation, is also highly effective for EDS, though its primary mechanism involves the consolidation and deepening of nocturnal sleep architecture, leading to improved daytime alertness (Mamelak 2022). In contrast, traditional psychostimulants, like methylphenidate, are now generally reserved as second‐ or third‐line therapies due to their less favorable side‐effect profiles including insomnia, cardiovascular effects, and potential for tolerance and dependence (Morton and Stockton 2000).

For the specific and often disabling symptom of cataplexy, as well as other manifestations of REM sleep intrusion like sleep paralysis and hypnagogic hallucinations, treatment focuses on agents that stabilize motor control and suppress REM sleep (Houghton et al. 2004). Sodium oxybate is widely considered the gold‐standard therapy, demonstrating profound efficacy in reducing the frequency and severity of cataplectic attacks (U.S. Xyrem Multicenter Study Group 2004). Pitolisant has also proven effective against cataplexy, offering a valuable therapeutic option that can simultaneously address both EDS and cataplexy with a single agent (Davis et al. 2021). In addition, REM‐suppressing antidepressants are frequently used off‐label. Among these, serotonin‐norepinephrine reuptake inhibitors (SNRIs) such as venlafaxine are particularly effective (Salin‐Pascual 2002). Their therapeutic action is attributed to the enhancement of monoaminergic tone in key brainstem nuclei, notably the locus coeruleus, which is critical for the regulation of REM sleep and the maintenance of muscle atonia, thereby preventing its intrusion into wakefulness (Schenck and Mahowald 2002).

## 7 Emerging Interventions and Future Directions

The therapeutic landscape for narcolepsy is poised for a paradigm shift, moving beyond purely symptomatic relief toward interventions that fundamentally target the disorder's underlying pathophysiology. At the forefront of this evolution is the concerted effort to restore the function of the hypocretin/orexin system, the loss of which is the defining feature of NT1 (Freeman et al. 2025). The most promising and clinically advanced of these strategies involves the development of selective orexin receptor 2 (OX2R) agonists. These small‐molecule agents, such as oveporexton and ORX750, are meticulously designed to mimic the effects of endogenous hypocretin, thereby offering the potential for a truly causal therapy rather than downstream modulation of wake‐promoting systems (Ishikawa et al. 2025). Clinical trial data have demonstrated their profound, dose‐dependent efficacy in improving wakefulness and, critically, in suppressing cataplexy, suggesting they can re‐establish physiological control over both sleep‐wake stability and REM sleep phenomena (Ishikawa et al. 2025). While this class of drugs represents a potential turning point for NT1 management, their development requires a cautious approach, as earlier candidates were halted due to adverse effects, underscoring the complexities of artificially replicating the nuanced role of the natural neuropeptide (Hartman et al. 2025).

Looking toward a more permanent solution, research is exploring the frontiers of gene and cell‐based therapies aimed at regenerating the source of hypocretin itself. Preclinical work is underway to introduce functional hypocretin‐producing genes into surviving hypothalamic neurons or to transplant stem cells engineered to differentiate into hypocretin‐secreting cells, though these ambitious restorative strategies remain in their infancy, facing significant technical and safety hurdles before human application (Kantor et al. 2013). In parallel, a distinct and equally critical line of investigation focuses on prevention through immunomodulation. Grounded in the strong evidence that NT1 is an autoimmune disorder, this approach seeks to intervene early in the disease course—ideally, soon after symptom onset—to halt the T‐cell‐mediated destruction of hypocretin neurons. The deployment of agents such as intravenous immunoglobulin (IVIG), corticosteroids, or targeted monoclonal antibodies could theoretically preserve the remaining neuronal population, altering the disease's trajectory (Giannoccaro et al. 2020). The ultimate success of this strategy, however, is fundamentally dependent on the discovery of reliable biomarkers that can enable a diagnosis within the narrow therapeutic window before irreversible neuronal loss occurs (Rach et al. 2025).

**FIGURE 3:**
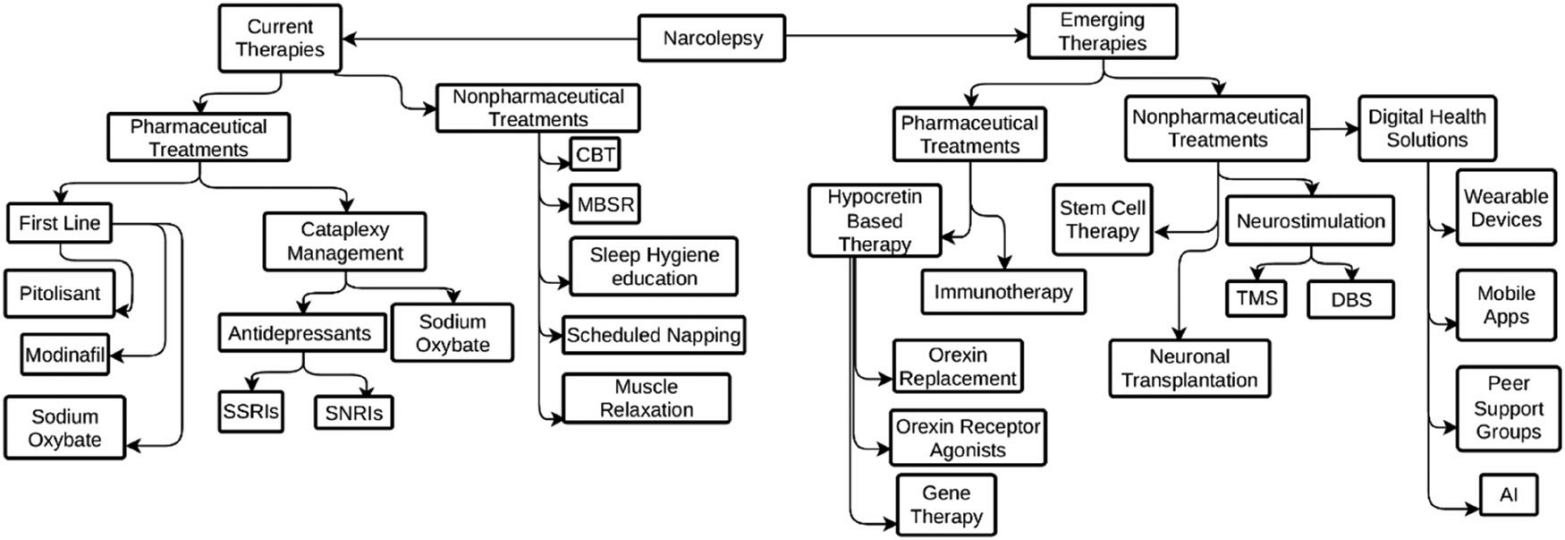
Flow chart of current and emerging therapies for narcolepsy.

Finally, at a broader systems level, advanced neuromodulation techniques are being refined to rebalance the dysregulated neural circuits that govern sleep and wakefulness. Non‐invasive methods like repetitive transcranial magnetic stimulation (rTMS), particularly when high‐frequency stimulation is applied over the left dorsolateral prefrontal cortex (DLPFC), have shown the ability to reduce EDS by enhancing cortical excitability in key attentional networks (Lai et al. 2017). More invasive approaches, such as deep brain stimulation (DBS), are also being explored for their potential to directly activate wake‐promoting centers in the brainstem and hypothalamus in the most refractory cases (Rogers et al. 2020). Together, these diverse and innovative research avenues—from molecular mimetics to cellular restoration, immune prevention, and circuit‐level modulation—form the comprehensive therapeutic pathway detailed in Figure 3, heralding a new, multimodal era for narcolepsy care where personalized strategies may finally modify the course of the disease itself.

## 8 Challenges and Limitations in Narcolepsy Management

Effective management of narcolepsy is profoundly constrained by a cascade of intersecting challenges that span from systemic diagnostic failures to the intricate complexities of long‐term therapy and patient adherence. The most significant and foundational barrier is the protracted diagnostic delay, which can stretch for eight to fifteen years from the initial onset of symptoms (Jung et al. 2025). This extensive gap is primarily driven by the heterogeneity of the clinical presentation and a pervasive lack of physician awareness, leading to frequent misattribution of symptoms to more common psychiatric disorders such as depression, anxiety, or schizophrenia (Ortiz et al. 2025). Consequently, by the time a correct diagnosis is established, the patient has often endured years of accumulating psychosocial damage, including diminished quality of life, academic underachievement, and professional setbacks (Maski et al. 2017). This creates an entrenched disease burden that complicates all subsequent therapeutic efforts. The problem is further exacerbated in many healthcare settings by limited access to the specialized tools essential for a definitive diagnosis, such as PSG and the MSLT, which delays or prevents accurate confirmation of the disorder (Jung et al. 2025).

Even after a diagnosis is secured, the therapeutic landscape itself presents formidable limitations. Current pharmacological interventions are primarily symptomatic rather than curative, offering partial relief from EDS and cataplexy without addressing the underlying loss of hypocretin neurons (Franceschini et al. 2020). While often effective, these medications carry a significant side effect profile, including headaches, nausea, and mood swings, which can deter long‐term use and compromise adherence (Thakrar et al. 2018). Moreover, some of the most potent therapies impose a substantial logistical burden on the patient; the strict and disruptive nightly dosing regimen required for sodium oxybate, for instance, demands a level of discipline that can be exceptionally difficult to maintain (Mayer et al. 2018). The management strategy is also frequently complicated by the high prevalence of psychiatric and metabolic comorbidities (Morse and Sanjeev 2018). The necessity of treating these co‐occurring conditions can create therapeutic dilemmas, as standard treatments for depression or hypertension may interact with narcolepsy medications or independently alter sleep architecture, muddying the clinical picture and challenging treatment optimization.

Finally, at the patient level, sustaining long‐term adherence is a central and multifaceted challenge (Finger et al. 2024). The high financial cost of many narcolepsy medications serves as a powerful and often insurmountable barrier, particularly in resource‐limited systems (Thorpy and Hiller 2017). Beyond pharmacology, the non‐pharmacological strategies that are crucial for comprehensive care—such as maintaining rigorous sleep hygiene and adhering to a schedule of prescribed daytime naps—require significant and sustained behavioral change. This demand for discipline is paradoxically imposed upon individuals whose capacity for self‐regulation is already compromised by the cognitive fog, fatigue, and motivational deficits characteristic of the disease itself (Harel et al. 2024). Overcoming these deeply interwoven barriers therefore requires a sophisticated, multimodal strategy that moves beyond simple prescription, integrating enhanced physician education with supportive tools like digital health applications and targeted psychological interventions such as cognitive‐behavioral therapy to empower patients and improve long‐term outcomes.

## 9 Conclusion

Narcolepsy remains a profoundly debilitating neurological disorder, where the lifelong burden on patients stands in stark contrast to a therapeutic landscape that, while evolving, still offers only symptomatic relief. The central challenge—and the field's greatest opportunity—lies in bridging the gap between managing the consequences of hypocretin neuron loss and directly addressing the underlying pathophysiology. Emerging interventions, particularly hypocretin receptor agonists and targeted immunotherapies, represent the vanguard of a crucial paradigm shift: Moving beyond mere symptom suppression toward the potential for genuine disease modification and restoration of neurological function. Translating this promise into clinical reality requires a concerted, multidisciplinary effort focused not just on novel molecules, but on forging the tools to validate them: Identifying reliable biomarkers for early diagnosis before irreversible psychosocial damage occurs, developing more comprehensive clinical endpoints that capture the disease's full 24‐h impact, and embedding patient‐centered outcomes into every stage of therapeutic development. Ultimately, the future of narcolepsy management lies not in incremental improvements to palliative care, but in advancing precision‐based, disease‐modifying interventions that can fundamentally alter the trajectory of the disorder and restore the potential for a full and unimpeded life.

## Author Contributions

**Natasha Elaine Hastings**: Conceptualized the review, defined the scope, and led manuscript drafting. **Khalil El Abdi**: Coordinated literature search and performed initial screening of sources. **Fazeela Bibi**: Synthesized relevant literature and contributed to analysis of findings. **Bilal Aslam**: Assisted with data organization, reference management, and manuscript editing. **Milka Rosario Nuñez**: Drafted key sections and participated in critical revisions. **Muhammad Tahla Rehman Sherani**: Helped develop review structure and contributed to methodology critique. **Sujeet Shadmani**: Participated in reviewing selected articles and integrating thematic evidence. **Umama Alam**: Assisted in drafting the introduction and background sections. **Vohra Maham Hassan**: Contributed to editing, formatting, and quality assurance. **Hoor‐e‐Ainaa**: Proofread the manuscript and helped ensure clarity and coherence. **Hania Imran**: Supported with summary tables and graphical elements. **James Hanna**: Provided critical manuscript revision and enhanced overall readability. **Said Hamid Sadat**: Supervised the review process, validated content, and approved the final submission. All authors reviewed and approved the final manuscript.

## Funding

The authors have nothing to report.

## Ethics Statement

The authors have nothing to report.

## Consent

The authors have nothing to report.

## Conflicts of Interest

The authors declare no conflicts of interest.

## Data Availability

This article is a narrative review, and as such, all data discussed are derived from previously published studies. The sources are fully available in the public domain and are comprehensively cited within the “References” section of this manuscript. No new datasets were generated or analyzed during the current study.
